# Vitamin D Insufficiency Is Common in Ugandan Children and Is Associated with Severe Malaria

**DOI:** 10.1371/journal.pone.0113185

**Published:** 2014-12-03

**Authors:** Sarah E. Cusick, Robert O. Opoka, Troy C. Lund, Chandy C. John, Lynda E. Polgreen

**Affiliations:** 1 Department of Pediatrics, University of Minnesota, Minneapolis, Minnesota, United States of America; 2 Department of Paediatrics, College of Health Sciences, Makerere University, Kampala, Uganda; 3 Department of Pediatrics, Los Angeles Biomedical Research Institute at Harbor, University of California Los Angeles, Los Angeles, California, United States of America; Centro de Pesquisa Rene Rachou/Fundação Oswaldo Cruz (Fiocruz-Minas), Brazil

## Abstract

Vitamin D plays an increasingly recognized role in the innate and adaptive immune response to infection. Based on demonstrated roles in up-regulating innate immunity, decreasing inflammation, and reducing the severity of disease in illnesses such as tuberculosis and influenza, we hypothesized that poor vitamin D status would be associated with severe malaria. We measured 25-hydroxyvitamin D [25(OH)D] by immunoassay in a sample of Ugandan children aged 18 months –12 years with severe malaria (cerebral malaria or severe malarial anemia, n = 40) and in healthy community children (n = 20). Ninety-five percent of children with severe malaria (n = 38) and 80% of control children (n = 16) were vitamin D-insufficient [plasma 25(OH)D <30 ng/mL]. Mean plasma 25(OH)D levels were significantly lower in children with severe malaria than in community children (21.2 vs. 25.3 ng/mL, p = 0.03). Logistic regression revealed that for every 1 ng/mL increase in plasma 25(OH)D, the odds of having severe malaria declined by 9% [OR = 0.91 (95% CI: 0.84, 1.0)]. These preliminary results suggest that vitamin D insufficiency may play a role in the development of severe malaria. Further prospective studies in larger cohorts are indicated to confirm the relationship of vitamin D levels to severity of malaria infection and to investigate causality.

## Introduction

In addition to its established role in maintaining calcium homeostasis and optimal bone health, vitamin D plays an important immune-modulating role as a key player in innate and adaptive immunity [1]. Poor vitamin D status can worsen infectious illness, as is perhaps best exemplified by its relationship with *M. tuberculosis*. Low plasma levels of vitamin D are associated with increased risk of active tuberculosis, while vitamin D supplementation accelerates recovery from the disease [2]–[3].

Work by Liu et al. [4] demonstrated that this benefit of vitamin D in tuberculosis is exerted via strengthening of the innate immune response to infection. Specifically, activation of toll-like receptor 2/1 (TLR 2/1) in human macrophages by mycobacterial surface lipoprotein up-regulates expression of the vitamin D receptor (VDR) and 1α-hydroxylase, the enzyme required to convert 25-hydroxy vitamin D [25(OH)D] to its active form, 1,25-dihydroxy vitamin D [1,25(OH)_2_D]. 1,25(OH)_2_D then binds to the VDR, increasing transcription of anti-microbial peptides (AMP), including cathelicidin, which results in improved response to anti-tuberculous therapy in individuals treated with vitamin D [5].

In addition to helping clear infection, vitamin D can also lessen disease severity by reducing inflammatory cytokines produced as by-products of immune activation. To date, this anti-inflammatory action of vitamin D and resulting improved clinical outcomes have been most clearly demonstrated with influenza, with a recent clinical trial reporting a decrease in pulmonary inflammatory response to influenza in individuals treated with 1,25[OH]_2_D [6]. In addition, mice treated with cathelicidin, an AMP upregulated by 1,25[OH]_2_D, have decreased inflammation and improved survival when exposed to influenza A as compared to untreated [7].

A potential beneficial role of vitamin D in malaria infection has not yet been well described. However, as with tuberculosis, the immune response to *Plasmodium falciparum* also includes activation of the innate immune system via toll-like receptors on macrophages [8], and cathelicidin was recently found to have anti-malarial properties *in vitro* [9]. Further, the pathogenesis of some forms of severe malaria, including cerebral malaria and severe malarial anemia, includes excessive levels of pro-inflammatory cytokines, including tumor necrosis factor – alpha (TNF-α), interferon-gamma (IFN-γ), and interleukin-6 (IL-6), all of which have been shown to be modulated by vitamin D [10]–[12].

Supporting a beneficial anti-inflammatory role of vitamin D in malaria, a recent study reported that among mice infected with *Plasmodium berghei* malaria, those that received oral supplementation with vitamin D did not develop experimental cerebral malaria [13]. Providing a mechanistic explanation for this finding, vitamin D significantly reduced circulating levels of IFN-γ and TNF-α, while increasing concentrations of interleukin-10 (IL-10) and regulatory T cells, and was associated with lower levels of cytoadherence molecules and better integrity of the blood-brain barrier.

To determine whether vitamin D status was associated with severe malaria in children, we determined 25(OH)D levels and the prevalence of vitamin D insufficiency in Ugandan children aged 18 month –12 years with severe malaria and in healthy community controls selected from the same household or village who were within one year of age. We hypothesized that the children with severe malaria would have lower levels of 25(OH)D, based on the evidence cited above that vitamin D may strengthen the innate immune response and protect against severe infection through decreased inflammation. A finding that vitamin D deficiency is more prevalent among children with severe malaria than among community children would suggest a potential role for vitamin D deficiency in the etiology of severe malaria and could lay the groundwork for future studies testing vitamin D supplementation as an adjuvant therapy for the condition.

## Methods

### Severe malaria pathogenesis study

From 2008–2013, we conducted a study of the pathogenesis of severe malaria and its neurocognitive outcomes at Mulago Hospital, Kampala, Uganda. Children with severe malaria [cerebral malaria (CM) or severe malarial anemia (SMA)] and healthy community children (CC) who were between 18 months and 12 years of age were enrolled and followed for two years to document neurobehavioral development.

The present analysis includes plasma samples collected at baseline from a subsample of 60 children [40 children with severe malaria (20 CM, 20 SMA) and 20 CC] enrolled in the larger study. These samples for the present analysis were randomly selected from the larger sample using a computer-generated algorithm in FileMaker Pro (Filemaker Inc., Santa Clara, CA).

For the parent study, CM was defined as: 1) coma (Blantyre Coma Score [BCS] ≤2 or Glasgow coma score [GSC]≤8); 2) *P. falciparum* on blood smear; and 3) no other known cause of coma. SMA was defined as *P. falciparum* on blood smear plus hemoglobin level ≤50 g/L. CC were within one year of age of children with severe malaria and were recruited from the same family or neighborhoods as children with CM or SMA. Eligibility criteria for CC were: 1) age 18 months to 12 years; 2) currently healthy; and 3) no illness requiring medical care within the previous four weeks. Known chronic illness was an exclusion criterion for all study groups.

### Sample collection and laboratory assessment

A venous blood sample of 5–7 mL was drawn into a heparinized blood collection tube from each child. Blood was centrifuged, and plasma was collected and stored at −80°C.

Plasma concentrations of 25(OH)D were measured by chemiluminescent immunoassay at the University of Minnesota Fairview Diagnostics Laboratory, Minneapolis, MN, a CLIA-certified laboratory. Vitamin D insufficiency was defined as a 25(OH)D level <30 ng/mL [14], and vitamin D deficiency was defined as a 25(OH)D level <20 ng/mL [14].

### Ethics statement

Written informed consent was obtained from parents or guardians of all study participants. Ethical approval was granted by the Institutional Review Boards for human studies at Makerere University School of Medicine, University of Minnesota, and Michigan State University.

### Statistics

We compared baseline socioeconomic, demographic, and clinical characteristics of CC and children with severe malaria using a t-test for normally distributed variables and Wilcoxon-rank sum test for non-Gaussian distributions. Proportions were tested using chi-square or Fisher’s Exact test when cell sizes were small (<5 children).

In unadjusted analysis, we first used a t-test to compare mean 25(OH)D in children with severe malaria to CC. We used ANOVA to test for differences in 25(OH)D level among the 3 groups, i.e., CM, SMA, and CC, and applied Tukey’s test to account for multiple comparisons. We then adjusted for potential confounders between malaria risk and vitamin D deficiency, including nutritional status (height-for-age z-score, weight-for-age z-score, and weight-for-height z-score), age, and sex, using multiple logistic regression with severe malaria as the outcome variable and 25(OH)D, anthropometric indices, age, and sex as predictor variables.

## Results

Community children and children with severe malaria were similar with regard to age, sex, height-for-age z-score, and indicators of socioeconomic status and food security (**Table 1**). Children with severe malaria had significantly lower hemoglobin (children with SMA had hemoglobin <50 g/L by study design) and also had significantly lower weight –for-height and weight-for-age z-scores than community children.

**Table 1:** Clinical and socioeconomic characteristics of study children.

	Severe Malaria	Controls	p-value^1^
n	40	20	
Age, years, mean (sd)	3.8 (1.7)	3.8 (2.0)	1.0
Male sex, no (%)	27 (67.5)	9 (45.0)	0.09
Hemoglobin, g/L, mean (sd)	52 (17)	114 (13)	<0.001
Height-for-age z-score, mean (sd)^2^	−0.43 (1.4)	−0.50 (1.5)	0.9
HAZ <−2 sd’s below reference median, no (%)	2 (10.0)	7 (18.9)	0.7
Weight-for-height z-score, mean (sd)^3^	−1.0 (1.9)	−0.05 (1.3)	0.03
WHZ <−2 sd’s below reference median, no (%)	8 (22.9)	0	0.05
Weight-for-age z-score, mean (sd)	−1.06 (1.4)	−0.40 (1.2)	0.03
WAZ <−2 sd’s below reference median, no (%)	8 (20.0)	0	0.05
Electricity in home, no (%)^4^	9 (25.7)	6 (30.0)	0.2
Family eats meat once per week, no (%)	20 (57.1)	11 (55.0)	0.9
Family has food year round, no (%)	32 (91.4)	19 (95.0)	0.6
Family owns radio, no (%)	22 (62.9)	15 (75)	0.4
Family owns bicycle, no (%)	5 (14.3)	3 (15.0)	0.9

1 P-value determined using t-tests for continuous variables, chi-square for proportions, and Fisher’s Exact for proportions with small sizes.

2 n = 37 for severe malaria group.

3 n = 35 for severe malaria group and n = 19 for control group.

4 n = 35 for severe malaria group for all SES and food security variables.

**Figure 1:**
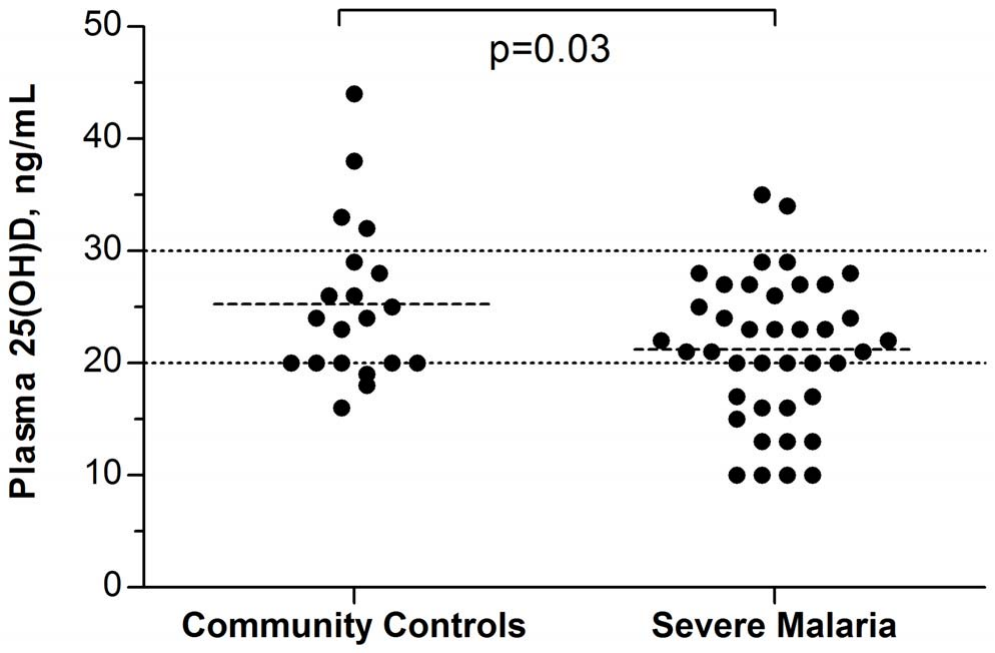
Unadjusted plasma 25(OH)D concentrations in Ugandan children with severe malaria (n = 40) and in healthy community children (n = 20). The dotted line at 30 ng/mL indicates the cutoff for vitamin D insufficiency, and the dotted line at 20 ng/mL indicates the cutoff for vitamin D deficiency. Dashed lines reflect mean 25(OH)D value for each study group.

Plasma concentrations of 25(OH)D were low overall and were significantly lower among children with severe malaria than among community children [mean (sd): 21.2 (6.4) ng/mL vs. 25.3 (7.1) ng/mL, p = 0.03 for unadjusted analyses, **Figure 1**]. Mean levels of 25(OH)D among CM children (21.3 ng/mL) and SMA children (21.2 ng/mL) were not statistically significantly different from each other (p = 1.0).

In total, 38 out of 40 (95%) children with severe malaria and 16 out of 20 (80%) CC were vitamin D insufficient (25(OH)D <30 ng/mL), and 12 out of 40 (30%) children with severe malaria and 3 out of 20 (15%) of CC were vitamin D deficient 25(OH)D <20 ng/mL). Of note, 20% of children with severe malaria, but no CC, had 25(OH)D levels below 15 ng/ml.

Logistic regression results revealed that for every 1 ng/mL increase in plasma 25(OH)D, the odds of having severe malaria declined by 9% [OR = 0.91 [95% CI: 0.84, 1.0]. This relationship did not change after controlling for weight-for-age z-score [adjusted OR: 95% CI = 0.91∶0.83, 1.0] or weight-for-height z-score [adjusted OR: 95% CI = 0.92∶0.84, 1.0].

## Discussion

In the present study, we found that vitamin D insufficiency is widespread among young Ugandan children and that 25(OH)D levels are significantly lower among children with severe malaria than otherwise healthy community children. To our knowledge, this is the first study to investigate vitamin D status in the context of malaria in children. The finding of an association between low 25(OH)D and severe malaria suggests a possible role of vitamin D deficiency in the etiology of severe malaria and establishes the foundation for larger studies. Confirmation of the findings of the present study would set the stage for testing vitamin D supplementation as a preventative or adjunctive therapeutic intervention for severe malaria.

The study finding that 80% of healthy community control children were vitamin D insufficient was surprising, particularly among a group of physically active children living near the equator and having year-round exposure to UVB radiation. Although the religious affiliation of the children in our study is not known, Uganda is 85% Christian and having children fully covered is uncommon. A recent study in Malawian infants and toddlers [15] found a mean 25(OH)D level of approximately 35.0 ng/mL, a full 10 ng/mL more than the mean of the control children in our study and nearly identical to the mean of 35.2 ng/mL reported among healthy toddlers in Boston [16]. The reason for the overall lower levels of 25(OH)D in our study population is unknown, but may include genetic variability in vitamin D binding protein [17], increased 25(OH)D degradation [14], or less direct sun exposure. It should be noted that dietary intake of vitamin D in this population is likely negligible. Vitamin D is not widely distributed in foods and occurs naturally only in some oily fish and shitake mushrooms. Unlike the United States, in Uganda, food products such as milk are not fortified with vitamin D, and vitamin D supplements are not widely available or affordable to this population.

While vitamin D levels were low overall, children with severe malaria have significantly lower 25(OH)D levels than community children. This finding was surprising and cannot be explained by differences in socioeconomic status or food security. To date, only one study has evaluated vitamin D levels over the course of a malaria infection [18]. In that study of malaria-infected adults, investigators found that 25(OH)D concentrations did not change over the course of acute malaria infection, i.e., from initial admission to the hospital to 14 days follow-up. This finding is important because it demonstrates that 25(OH)D is not affected by severe malarial infection and thus remains a reliable marker of vitamin D status even in individuals with significant inflammation.

A recent study proposed that vitamin D binding protein must be considered when assessing vitamin D status [17]. In that study of more than 2000 American adults, black individuals had significantly lower 25(OH)D concentrations than their Caucasian counterparts, but they also had lower concentrations of vitamin D binding protein, resulting in similar concentrations of free, and thus bioavailable, 25(OH)D according to the study’s authors. Bioavailable 25(OH)D was determined using a formula that took into account the prevalence of the two most common polymorphisms of the vitamin D binding protein gene. These study findings raise important issues, but a number of concerns have been noted about the study’s laboratory methods, extrapolation from the bioavailability formula, and the authors’ overall interpretation of results [19]. Thus the overall significance of vitamin D binding protein levels in individuals of African descent remains unclear. Nevertheless, future studies of vitamin D status in malaria-endemic or other areas would likely benefit from concurrent measurement of vitamin D binding protein, as well as other functional indicators such as parathyroid hormone, so that full investigation of the association of these markers with important clinical outcomes can be determined.

Limitations to our study include the small sample size and the cross-sectional design, which limits our ability to make any conclusions regarding a causal effect of 25(OH)D level on severity of malaria. The study findings should be viewed as preliminary data, but the finding that 25(OH)D was significantly lower among children with severe malaria than among community children merits further investigation. Future studies should assess vitamin D levels in a larger cohort of children, and test whether vitamin D levels in these children relate to production of anti-microbial peptides, such as cathelicidin, via a mechanism analogous to that observed with *M. tuberculosis,* and to level of inflammatory response to malaria.

In conclusion, we found that vitamin D insufficiency is common in Ugandan children, that children with severe malaria have significantly lower levels of 25(OH)D than healthy community children, and that lower levels of vitamin D are associated with increased odds of severe malaria. Future studies investigating a possible role for vitamin D in mounting an optimal immune response to malaria and in protecting from severe disease are warranted.

## Supporting Information

Data S1 (XLSX)
